# Rapid and Simultaneous Detection of Major Drug Resistance Mutations in Reverse Transcriptase Gene for HIV-1 CRF01_AE, CRF07_BC and Subtype B in China Using Sequenom MassARRAY® System

**DOI:** 10.1371/journal.pone.0153641

**Published:** 2016-04-19

**Authors:** Ka-Wai Cheung, Qiaoli Peng, Liufen He, Kanru Cai, Qiang Jiang, Boping Zhou, Sabrina Wai-Chi To, Wing-Cheong Yam, Li Liu, Zhiwei Chen, Hui Wang

**Affiliations:** 1 AIDS Institute and Department of Microbiology, Research Centre for Infection and Immunity, Li Ka Shing Faculty of Medicine, The University of Hong Kong, 21 Sassoon Road, Pokfulam, Hong Kong SAR, People’s Republic of China; 2 HKU-AIDS Institute Shenzhen Research Laboratory and AIDS Clinical Research Laboratory, Guangdong Key Lab of Emerging Infectious Diseases and Shenzhen Key Lab of Infection and Immunity, Shenzhen Third People’s Hospital, Guangdong Medical College, Shenzhen, People’s Republic of China; 3 Department of Microbiology, The University of Hong Kong, Hong Kong SAR, People’s Republic of China; University of Malaya, MALAYSIA

## Abstract

The development of a rapid, high-throughput and cost-effective HIV-1 drug resistance (HIV-DR) testing system is a challenge for areas consisting different HIV-1 strains. In this study, we established a broadly reactive multiplex assay that could simultaneously detect major drug resistance mutations at 8 loci, which are associated with resistance to commonly used nucleoside reverse transcriptase inhibitors (NRTIs) and Non-nucleoside reverse transcriptase inhibitors (NNRTIs), in specimens of HIV-1 CRF01_AE, CRF07_BC and subtype B, the three major circulating strains in China, using the matrix-assisted laser desorption ionization-time of flight mass spectrometry (MALDI-TOF MS) provided by Sequenom MassARRAY® system. To establish the assay, *pol* gene fragments were prepared from the plasma viral RNA of 159 patients by nested PCR and the presence of wild type and mutant alleles at the 8 loci were analyzed by MALDI-TOF MS. In terms of loci, the detection rate of the alleles was greater than 97% for M41L, K65R, M184V and G190A, 91.2% for K101E/Q/P, 91.2% for T215F/Y, 89.9% for K103N/S and 80.5% for L210W. In terms of individuals, 80% of the alleles were detected in 95.4% CRF01_AE patients, 100% CRF07_BC patients and 83.3% subtype B patients. Importantly, the MALDI-TOF MS results were concordant to the drug resistance profiles of patients obtained from conventional sequencing analysis after excluded the failed detections. Using plasmid templates, the assay was estimated to be sensitive to detect drug resistant variants at level about 20% of the circulating viral population. The capability of this assay to detect mixed viral populations was further verified by two different patient specimens. In conclusion, this study evaluated the use of Sequenom MassARRAY® system for high-throughput detection of HIV-DR mutations towards the commonly used reverse transcriptase inhibitors in China.

## Introduction

Over the past 3 decades, HIV-1/AIDS has become one of the world’s leading infectious diseases and has already caused over 30 million deaths throughout the world. The development of antiretroviral drugs and implementation of highly active antiretroviral therapy (HAART) have remarkably reduced the morbidity and mortality caused by HIV-1 infection [1]. Despite the dramatic reduction of plasma HIV-1 level in patients on HAART, the virus is not eradicated and a lifelong treatment is required. Apart from the side effects, drug resistant HIV-1 evolved under the selective pressure imposed by antiretroviral drugs after long-term treatment probably leads to clinical manifestation. To date, drug resistant HIV-1 has been readily found and is transmitting in many countries where antiretroviral treatment is provided [2,3].

Based on the international guidelines for antiretroviral treatment, drug resistance test is recommended before HAART and in patients of confirmed virologic failure [4]. Current drug resistance assays can be divided into genotypic assay and phenotypic assay. Genotypic assay bases on the determination of specific point mutations on the nucleotide sequences of the patient’s virus whereas phenotypic assay bases on measuring the replication of virus isolates from patients in the presence or absence of drugs. The major difference between these two methods is that genotypic assay can generate resistance profiles in a faster, more informative and cost effective way.

Conventional sequencing based assays including ViroSeq [5] and TruGene [6] as well as many in-house assays [7,8] are widely adopted for genotypic drug resistance testing in clinical settings. However, these assays are often not sensitive enough and are labor-intensive for the detection of the low abundance mutations. Sequenom MassARRAY® system is a DNA analysis platform which has been widely utilized for high-throughput genotyping studies such as single nucleotide polymorphism (SNP) detection [9–12] based on the matrix-assisted laser desorption ionization-time of flight mass spectrometry (MALDI-TOF MS). In the context of HIV-1, a change from wild type allele to mutant allele in a virus would result in products with mass differences from the wild type strain after primer-extension reactions and therefore enables drug resistance detection by MALDI-TOF MS.

Due to the mounting evidence that antiretroviral therapy is beneficial to HIV-1 prevention and the increasing emergence and transmission rate of drug resistant HIV-1 [1–3,13], the demand for rapid, high-throughput and cost-effective drug resistance testing system is significantly elevated, especially in areas where limited antiretroviral drug variety supplied. Also, there are only few multiplex assays that can detect drug resistance mutations for various HIV-1 subtypes simultaneously [14]. In this study, we established a multiplex assay for detecting the drug resistance mutations at 8 loci (M41L, K65R, K101E/Q/P, K103N/S, M184V, G190A, L210W and T215F/Y), which are associated with resistance to commonly used nucleoside reverse transcriptase inhibitors (NRTIs) and Non-nucleoside reverse transcriptase inhibitors (NNRTIs) based on the automated MassARRAY® system (Sequenom). The assay was validated in 159 patients mainly infected with HIV-1 CRF01_AE, CRF07_BC and subtype B, the three major circulating strains in China [15,16]. This study provided a technical base and evaluated the potential use of Sequenom MassARRAY® system for high-throughput detection of HIV-1 drug resistance (HIV-DR) mutations in China.

## Materials and Methods

### Specimens

159 plasma specimens were collected between 2011 and 2013 from HAART experienced or treatment naïve HIV-1 infected patients at the AIDS clinic of Shenzhen Third people’s Hospital after written informed consent was obtained from each participant. This study was conducted in compliance with the Declaration of Helsinki and was approved by the ethics review committee of Shenzhen Third people’s Hospital. The drug resistance profiles of all specimens were undetermined at the time of collection. The demographic characteristics of the specimens were summarized in Table 1.

**Table 1:** Demographic characteristics of 159 HIV-1-infected patients.

Characteristic	Value
Mean age in years (range)	36.3(2–67)
No. male (%)	130(81.8)
No. female (%)	29(18.2)
Risk factor for HIV infection (% of patients)	
Heterosexual	79(49.7)
Homosexual	65(40.9)
Vertical transmission	1(0.6)
Intravenous drug	3(1.9)
Unknown	11(6.9)
WHO clinical stage (%)	
I	35(22.0)
II	48(30.2)
III	1(0.6)
VI	63(39.6)
Unknown	12(7.5)
Subtyping (%)	
CRF01_AE	109(68.6)
CRF07_BC	26(16.4)
CRF08_BC	2(1.3)
B	12(7.5)
URF_01/BC	3(1.9)
CRF02_AG	1(0.6)
URF_01/C	1(0.6)
Undetermined	5(3.1)
Mean CD4 cell count (No. of cells/μl [range]) ^a^	208(1–1663)
No. of unknown (%)	6(3.8)
Median HIV-1 RNA (log_10_ copies/ml)(interquartile range) ^b^	5.7(5.1–6.3)
No. of HIV-1 <500 copies/ml (%)	4(2.5)
History of actual treatment (No. of patients) (%)	
No ART	126(79.2)
With NRTI	1(0.6)
With NRTI plus NNRTI	24(15.1)
With NRTI plus PI	1(0.6)
With NRTI plus NNRTI plus PI	7(4.4)

NRTI, nucleoside reverse transcriptase inhibitor: lamivudine, tenofovir, stavudine, zidovudine; NNRTI, non-nucleoside reverse transcriptase inhibitor: efavirenz, nevirapine; PI, protease inhibitor: ritonavir, lopinavir

^a^: The CD4 cell count of the 2 years old patient (No. of cells/μl: 1078) is excluded

^b^: The viral load of the 4 patients with HIV-1 copy number less than 500 copies/ml is not included

### RNA extraction, cDNA synthesis, amplification of first round PCR products of the nested PCR and viral load determination

5ml peripheral blood from each patient was collected into red-top tubes containing anticoagulant. After centrifugation, 2ml plasma was collected and stored at -80°C. HIV-1 viral RNA was extracted from plasma with the High Pure Viral RNA kit (Roche) according to manufacturer’s instruction. cDNA synthesis and the amplification of first round PCR products of the nested PCR were prepared by PrimeScript^TM^ one step RT-PCR kit Ver.2.0 (Takara) following the manufacturer’s instruction. In detail, the reaction mixture (25μl) for cDNA synthesis and first round PCR contained 5μl extracted viral RNA (4–25μg/ml) and 0.4μM first round PCR primers. The viral load of each specimen was determined by careHIV-1 RT-PCR Assay (Qiagen) and analyzed by Lightcycler (Roche) following the manufacturer’s instruction.

### Extension primer design and detection of drug resistance mutations in the *pol* gene fragment of HIV-1

The drug resistance mutations towards reverse transcriptase inhibitors in the *pol* gene fragment were detected by MALDI-TOF MS using Sequenom massARRAY® system. The extension primers for detecting the wild type and the drug resistant mutant alleles in the *pol* gene fragment were designed by MassARRAY® Assay Design 3.1 software. All the primers were purchased from Tech Dragon Limited and other reagents were purchased from Sequenom unless otherwise specified.

### 4 main steps for the detection of HIV-1 variants by MALDI-TOF MS

#### Nested PCR to amplify the *pol* gene fragment

The *pol* gene fragment with size around 1257bp including the protease gene and the first 313 codons of reverse transcriptase gene was amplified using nested PCR method. The first round PCR was performed with primer pairs PR/RT F1 (sense: 5’-YYC AgA gCA gAC Cag AgC CAA C -3’) and PR/RT R1 (antisense: 5’- CYT gCC AAT AST CYR TCC ACC-3’) and the second round PCR was performed with primer pairs PR/RT F2 (sense: 5’- CTT CCC TCA GAT CAC TCT TTG GC -3’) and PR/RT R2 (antisense: 5’- GCT CTT GAT AAA TTT GGT ATG TCC ATT G -3’). The first round PCR products were directly generated from viral RNA using PrimeScript^TM^ one step RT-PCR kit Ver.2.0 as mentioned above at thermal cycling conditions: 50°C for 30min, 94°C for 2min, followed by 30 cycles of 94°C for 30s, 58°C for 30s, and 72°C for 1.5min, with a last extension step of 72°C for 7min. The second round PCR was conducted by using PrimeSTAR (Takara) with thermo cycling conditions: 98°C for 3min, followed by 30 cycles of 98°C for 10s, 60°C for 5s, and 72°C for 1min, with a last extension step of 72°C for 7min.

#### Shrimp Alkaline phosphatase (SAP) Treatment

The unincorporated dNTP in the PCR reaction was neutralized by SAP solution provided in the SpectroCHIP Arrays and Clean resin kit according to the manufacturer’s instruction (Sequenom). In brief, 4μl of PCR product was first aliquoted into 384 wells plate, mixed with 2μl SAP solution followed by incubating in 37°C for 40 min. The reactions were stopped by inactivation at 85°C for 5 min.

#### Multiplex primer extension reactions

The 8 selected drug resistance mutations towards reverse transcriptase inhibitors were analyzed in 6 separate primer-extension reactions (6-plex in assay 1, 5-plex in assay 2, 4-plex in assay 3, 4-plex in assay 4, 2-plex in assay 5, 1-plex in assay 6). The sequences and molecular weights of extension primers and extension products were listed in S1 Fig. The reaction was performed following the manufacturer’s instruction.

#### Sample conditioning, Data acquiring and analysis

The extension products obtained were desalted by adding 16μl double distilled water and 6mg SpectroCLEAN resin before MALDI-TOF MS analysis. The 384 wells plate containing the samples was centrifuged at 360g for 5 min before transferring the samples to the SpectroCHIP via the MassARRAYnano dispenser. Data from SpectroCHIP was acquired by MassARRAY® System (Sequenom) and analyzed by TyperAnalyzer Application, version 4.0 (Sequenom).

### TA cloning

*Pol* gene fragment was amplified using Taq polymerase (Takara) with the first round PCR products served as template. The amplified PCR product was then cloned into TA cloning vector (Takara) and transformed into *Escherichia coli* (E.coli) cells (DH5α) according to the manufacturer’s instruction. Transformed cells were selected on a LB agar plate containing 100μg/ml ampicillin at 37°C for 16–18 hours. Positive transformants were picked and inoculated into 2ml LB broth containing 100μg/ml ampicillin for plasmid propagation. Successful ligation was verified by PCR using second round PCR primers for PR/RT region as mentioned and DNA sequencing.

### Plasmid isolation and construction of plasmid containing M184V mutation

Plasmid was isolated from E.coli (DH5α) using mini-prep DNA purification kit (Qiagen) according to the manufacturer’s instruction. Purified plasmid was then subjected to site-directed mutagenesis using the QuikChange II Site-Directed Mutagenesis kit (Agilent technologies) for the construction of 184V mutant. Successful mutagenesis was verified by PCR amplification of the *pol* gene fragment followed by DNA sequencing.

### DNA sequencing

All the PCR products were sequenced at Beijing Genomic Institute-Shenzhen (BGI-Shenzhen). The sequencing primers used were the same as the primer pairs PR/RT F2 and PR/RT R2 as mentioned above.

### Genotypic analysis of HIV-1 drug resistance in patient specimens

Sequence data of the *Pol* gene fragment of each patient specimen obtained from DNA sequencing was submitted to Stanford University HIV drug resistance database for generating an antiretroviral drug resistance profile [17].

## Results

### Amplification of the *pol* gene fragment from HIV-1 CRF01AE, CRF07_BC and subtype B for drug resistance mutation detection

**Fig 1:**
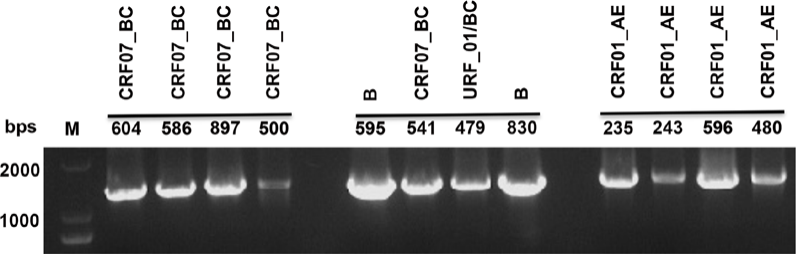
Amplification of *pol* gene fragment by nested PCR. Viral RNA extracted from plasma specimens of patients was first converted to cDNA and the *pol* gene fragment was amplified by nested PCR. Result of gel electrophoresis illustrated the nested PCR method was capable to amplify the *pol* gene fragment with high specificity from HIV-1 of various strains as indicated.

The vast genetic variation among HIV-1 isolates often increases the difficulties for the development of assay platforms for genotypic drug resistance assay, especially the one for multiplex detection. In order to detect alleles responsible for HIV-DR mutations towards reverse transcriptase inhibitors as well as to increase the efficiency and efficacy of our assay, we adapted the nested PCR method as described previously [18]. Result in Fig 1 illustrated the nested PCR method in this study was capable to amplify the *pol* gene fragment with high specificity from HIV-1 CRF01_AE, CRF07_BC and subtype B. The primers also worked for the amplification of same region from HIV-1 of other subtypes, such as URF_01/BC. The PCR products were confirmed to be the desired region by DNA sequencing.

### Performance evaluation by using clinical specimens

To evaluate the performance of the MALDI-TOF MS based assay, we analyzed plasma specimens from 159 patients. The demographic characteristics of these patients were summarized in Table 1. In this study, the performance of the assay was defined in 2 contexts as described previously [10]: 1) the call rate per allele, which was the percentage of successful call for an indicated allele among all specimens, and 2) the call rate per sample, which was the percentage of alleles successfully called in each individual specimen.

Firstly, we considered the call rate per allele. As shown in Table 2, 9 out of 10 alleles except L210W achieved a call rate greater than 90%. The overall call rate for all alleles in all 159 specimens was 93.8%. More importantly, the call rate per allele for codon 65, 184 and 190, which were the 3 most common drug resistance mutation sites found among our specimens, were greater than 97%. Since 2 simultaneous point mutations were required for converting the lysine (K) into glutamic acid (E), glutamine (Q) or proline (P) in codon 101 and asparagine (N) or serine (S) in codon 103, 2 alleles were required to be called simultaneously in order to give conclusive results for K101E/Q/P and K103N/S. According to our result, the 2 pairs of alleles were called simultaneously in 145 out of 159 specimens for K101E/Q/P (91.2% of the total specimens) and in 143 out of 159 specimens (89.9% of the total specimens) for K103N/S.

**Table 2:** Summary of the call rate per allele.

	Allele detected at each site ^a^	Call rate per allele (%)	Combined call rate for K101E/Q/P and K103N/S (%)
**M41L**	**[A/c/t]TG**	97.5	
**K65R**	**A[A/g]A**	100	
**K101E/Q/P**	**[A/g/c]AA**	91.2	91.2
**K101E/Q/P**	**A[A/c]A**	98.7	
**K103N/S**	**A[A/g]A**	90.6	89.9
**K103N/S**	**AA[A/G/t/c]**	91.2	
**M184V**	**[A/g]TG**	97.5	
**G190A**	**G[G/c]A**	99.4	
**L210W**	**T[T/g]G**	80.5	
**T215F/Y**	**[A/t]CC**	91.2	
**Overall call rate for all 1590 alleles (%)**	** **	93.8	

^a^ Alleles to be detected are shown in the brackets and the small letters represent the mutant alleles

**Fig 2:**
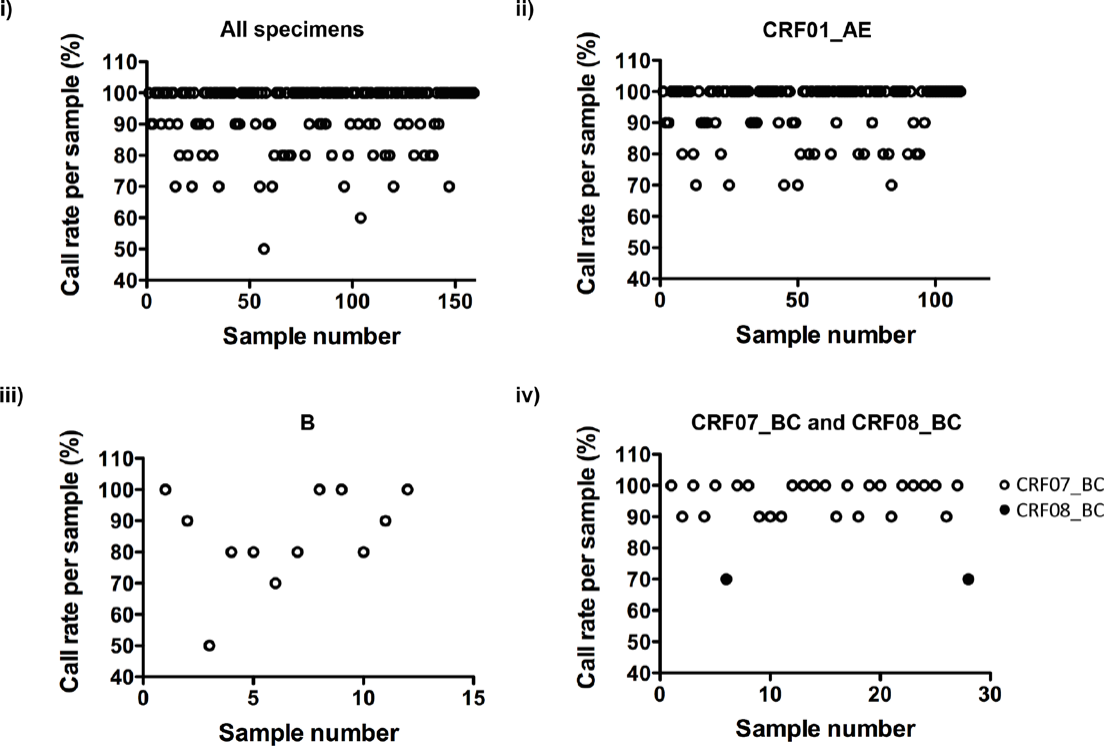
Summary of call rate per sample for the 159 clinical specimens A summary of call rate per sample for the total 159 clinical specimens and specimens of HIV-1 CRF01_AE, subtype B, CRF07_BC and CRF08_BC are demonstrated. i) The total call rate per sample of all 159 clinical specimens; ii) The call rate per sample of 109 specimens of CRF01_AE; iii) The call rate per sample of 12 specimens of subtype B and iv) The call rate per sample of 26 specimens of CRF07_BC and 2 specimens of CRF08_BC.

The call rate per sample was subsequently evaluated in order to examine the effectiveness for clinical application. As shown in Fig 2I, 130 out of 159 (81.8%) specimens had a call rate greater than 90%, 149 out of 159 (93.7%) specimens had a call rate greater than 80%. We further evaluated the call rate per sample for specimens of HIV-1 CRF01_AE, subtype B, CRF07_BC and CRF08_BC. For CRF01_AE, 90 out of 109 (82.6%) specimens had a call rate greater than 90%, and 104 out of 109 (95.4%) specimens had a call rate greater than 80%. For subtype B, 6 out of 12 (50%) specimens had a call rate greater than 90%, and 10 out of 12 (83.3%) specimens had a call rate greater than 80%. For CRF07_BC, 26 out of 26 (100%) specimens had a call rate greater than 90% and the call rate of the 2 CRF08_BC specimens were 70%. Results were demonstrated in Fig 2II, 2III and 2IV respectively. The discrepancies of the call rate per sample shown in Fig 2 were found to be mainly due to the genetic variations of HIV-1 among specimens after sequence alignment. Other possible factors for the discrepancies included the concentration of virus in the specimens and the quality of the PCR products. Although the call rate per sample was not completely ideal, the assay was able to detect major resistance mutations (M41L, K65R, M184V and G190A) in 150 out of 159 (94.3%) specimens simultaneously.

Apart from the call rates, the reproducibility of the assay was evaluated. The reproducibility of the platform was examined by amplifying the *pol* gene fragments from the same plasma specimens of 12 patients. Interestingly, we found that all of the 10 alleles detected in these patients were the same as those identified in the first time after excluded the failed detections (data not shown). This result suggested the MS platform established was consistent and stable enough.

### Estimation of the sensitivity for detecting drug resistant variants in mixed viral populations

**Fig 3:**
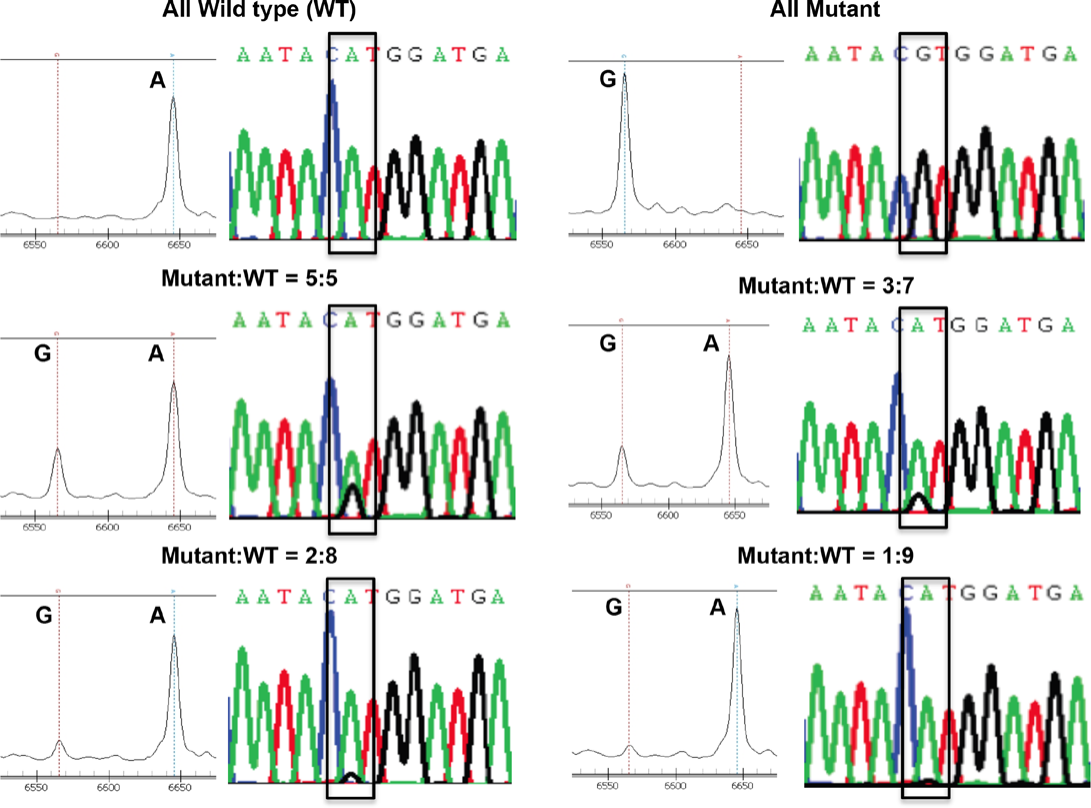
Evaluation of the sensitivity of the MALDI-TOF MS for detection of mixed genotypes. Results of MALDI-TOF MS and direct sequencing for the PCR products obtained from different mixtures of the wild type plasmid (M184) and the mutant plasmid (184V) are demonstrated. The ratio of the plasmids in the mixtures are as indicated. The 184V mutant could be detected when the ratio of mutant plasmid to wild type plasmid was as low as 2 to 8 in both MALDI-TOF MS and direct sequencing. The site of mutation is indicated by a box in the sequencing chromatogram.

Since drug resistant HIV-1 may exist in very low frequency, it is necessary to understand the sensitivity of this MALDI-TOF MS based assay for determining the presence of low abundance mutant virus in mixed viral populations. To evaluate the sensitivity of this assay, a total of 100ng plasmid mixture was first prepared by mixing plasmids carrying wild type (WT) genotype (M184 in the RT region) and plasmids carrying mutant genotype (184V in the RT region) in ratios as indicated in Fig 3. The mixtures were then subjected to nested PCR and analyzed by the MALDI-TOF MS. Result in Fig 3 demonstrated the 184V mutant could be detected when mutant to wild type ratio was as low as 2 to 8 (20%). This result was close to the clinical and other conventional genotypic assays, which detected drug resistant variants at level about 20% of the circulating viral population [6,19].

### Validation by DNA sequencing and Stanford University HIV drug resistance database

**Fig 4:**
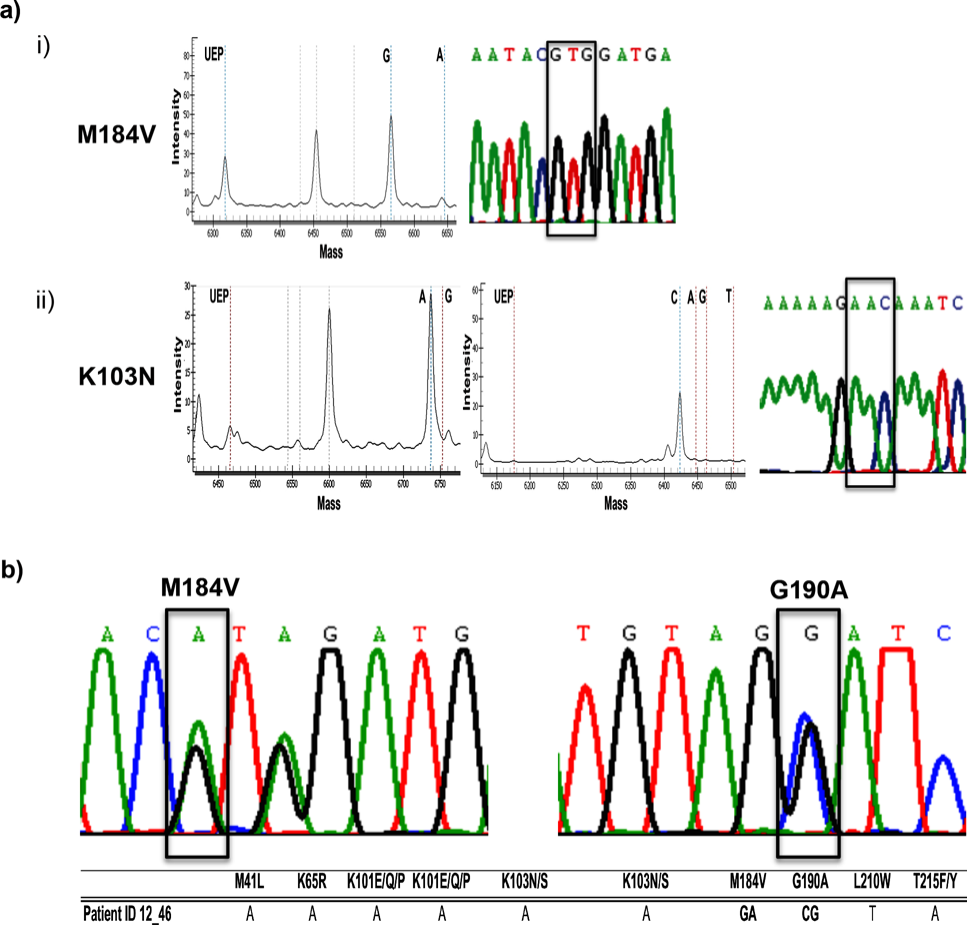
Comparison of the results between the MALDI-TOF MS and direct sequencing. Results of MALDI-TOF MS and direct sequencing for the PCR products obtained from clinical specimens are demonstrated. a) i) A peak at molecular mass 6565.3 indicated a A (M184) to G (184V) mutation in codon 184 of the RT region. ii) A combined result of peaks at molecular mass 6736.5 (left) and 6423.3 (middle) indicated a AA (K103) to AC (N103) mutation in codon 103 of the RT region. The codon is indicated by a box in the sequencing chromatogram. b) The mixed genotypes at codon 184 and 190 detected by MALDI-TOF MS as shown in the table were concordant to the sequencing chromatogram. The specific site of mixed genotype is indicated by a box.

To validate the MALDI-TOF MS result, the same PCR products of the 159 specimens used for MALDI-TOF MS analysis were sequenced by conventional DNA sequencing. 154 out of 159 specimens were sequenced successfully and the sequences were submitted to Stanford University HIV drug resistance database for generating the antiretroviral drug resistance profile of each specimen. The drug resistance profiles generated from the sequences of drug resistant specimens were concordant to the MALDI-TOF MS results except patient 12_46, after excluded the failed detections. Indeed, the virus of this patient had a 184V mutation in the *pol* gene fragment when the sequencing chromatogram was carefully analyzed (Fig 4B). A comparison of the MALDI-TOF MS results and the drug resistance profiles of drug resistant specimens from the database is shown in Table 3. Representative results demonstrating the concordance between the chromatograms of DNA sequencing and the MALDI-TOF MS results at two different drug resistance mutation sites (M184V and K103N) are shown in Fig 4A.

**Table 3:** Comparison of the HIV-DR mutations obtained from MALDI-TOF MS and Stanford HIV-DR database.

Specimen ID	Subtyping	Mutations detected by MALDI-TOF MS	Drug resistance profile shown on the Stanford drug resistance database
12_102	CRF01_AE	M184V, G190A	**M184V, G190A**
3_328	CRF01_AE	M184V	V75L, V106M, Y115F, V179D, **M184V**
28_139	CRF01_AE	M184V, G190A	**M184V, G190A**
8_42	CRF01_AE	K65R	**K65R**, V179D, Y181C
904	CRF01_AE	M184V	**M184V**
A654	CRF01_AE	M184V, G190A	V106M, **M184V**, **G190A**
676	CRF01_AE	K101E/Q/P, G190A, Failed to call allele for M184V	**K101E**, **M184V**, **G190A**
22_326	CRF01_AE	K65R, K103N/S	**K65R**, **K103N**, V106M
12_46	CRF01_AE	M184V, G190A	Y181C, M184I, **G190A**
25_13	B	M184V, Failed to call both alleles for K103N/S	V90I, **K103N**, **M184V**, K238N
9_636	B	K103N/S	**K103N**
10_774	B	K65R	**K65R**
478	CRF07_BC	M184V	**M184V**
16_559	CRF07_BC	M184V	D67G, K70E, V106M, Y115F, V179D, **M184V**, F227L
824	CRF08_BC	K65R, M184V	**K65R,** Y181C, **M184V**, G190C, H221Y

In order to examine any discrepancies between the MALDI-TOF MS results and conventional DNA sequencing results, results of the 154 specimens from both assays were compared. In total 1540 alleles detected by both assays, 1446 (93.9%) were identical and 94 (6.1%) were discordant. The discrepancies between the two assays were due to the failed detections by the MALDI-TOF MS. Analysis of the DNA sequencing results revealed the MALDI-TOF MS based assay missed these alleles mainly due to mismatches between the extension primers and the viral sequences. The concordance rates between the results of the MALDI-TOF MS and the DNA sequencing at each drug resistance mutation site in 154 patient specimens were summarized in Table 4.

**Table 4:** Concordance between MALDI-TOF MS and conventional DNA sequencing at each allele in 154 specimens.

	Allele detected at each site ^a^	No. of allele detected by MALDI-TOF MS	No. of failed detections by MALDI-TOF MS	No. (%) of specimens with concordant results
M41L	[A/c/t]TG	150	4	150 (97.4)
K65R	A[A/g]A	154	0	154 (100)
K101E/Q/P	[A/g/c]AA	141	13	141 (91.6)
K101E/Q/P	A[A/c]A	152	2	152 (98.7)
K103N/S	A[A/g]A	140	14	140 (90.9)
K103N/S	AA[A/G/t/c]	140	14	140 (90.9)
M184V	[A/g]TG	151	3	151 (98.1)
G190A	G[G/c]A	153	1	153 (99.4)
L210W	T[T/g]G	125	29	125 (81.2)
T215F/Y	[A/t]CC	140	14	140 (90.9)
Total		1446	94	1446 (93.9)

^a^ Alleles to be detected are shown in the brackets and the small letters represent the mutant alleles

Conventional DNA sequencing method for identifying the presence of mixed viral genotypes from patient specimens is time consuming and labor intensive. Although some computer programs can facilitate the sequence analysis, identification of low abundance variants by these programs may be affected by the noise from the background of sequencing results. Alternatively, the presence of low abundance variants may be missed if the sequencing results were analyzed manually. As a result, there is a great demand for a rapid method for identifying mixed viral populations in patients in order to allow doctors to arrange the treatment regimens and to prevent the transmission of drug resistant strains. Interestingly, the assay used in this study was able to detect mixed viral populations in two different patient specimens and the results were concordant to the chromatogram obtained from DNA sequencing. One of the representative results is shown in Fig 4B. Of these two specimens, no drug resistance mutation was found in one sequence (data not shown) while the other one (patient 12_46), as shown in Fig 4B, no 184V mutation was identified after submitted both sequences to Stanford University HIV drug resistance database for analysis. These results further validated the ability of this MALDI-TOF MS based platform for the detection of mixed viral populations.

## Discussion

The nationwide “Comprehensive AIDS Response Program” launched by Chinese government provides free HAART for all AIDS patients living in rural areas and patients with financial difficulties living in urban districts [20,21]. Since the China’s National Free Antiretroviral Treatment Program (NFATP) began in 2002, ART usage has scaled up rapidly. By the end of 2012, more than 208,216 patients across the country had received free antiretroviral treatment [22]. Many studies found that the NFATP has successfully increased life expectancy and reduced mortality among Chinese HIV patients [23–25]. Although rapid ART scale-up significantly reduced AIDS-related morbidity and mortality, HIV antiretroviral treatment programs are facing the challenge of potential widespread emergence and transmission of drug resistant HIV-1 [26–30]. As a result, it is necessary to establish a rapid drug resistance testing system in order to assure the efficacy of the antiretroviral therapy.

In this study, we described the development and validation of a broadly reactive MALDI-TOF MS based assay for multiplex detection of drug resistance mutations towards the commonly used NRTIs and NNRTIs for various HIV-1 strains, mainly CRF01_AE, CRF07_BC and subtype B in China. Specifically, this assay could simultaneously detect mutations associated with drug resistance towards ABC, AZT, d4T, 3TC, ddI, TDF, NVP and EFV, which are the reverse transcriptase inhibitors freely provided by the Chinese government and are used in many developing countries in the world [31].

In comparison with recently reported multiplex genotypic HIV-1 drug resistance assays [14,32], our assay showed broad reactivity in detecting drug resistance mutations for HIV-1 CRF01_AE, CRF07_BC and subtype B although fewer drug resistance mutations were investigated. Our assay also worked for other HIV-1 strains such as URF_01/BC and CRF02_AG (data not shown). Despite the relative low call rate per sample for the two CRF08_BC specimens as shown in Fig 2IV, our assay was able to detect drug resistance mutations in one of the CRF08_BC specimens as shown in Table 3. More specimens will be needed for evaluating the efficiency of our assay for specimens of CRF08_BC in future. Indeed, the broad spectrum of detection is of significant importance in areas consisting different HIV-1 strains and in clinics where the genotypic information of the virus is always unavailable.

One of the greatest challenges for our assay is the genetic variation among different HIV-1 strains. As a result, we considered alleles with call rate per allele greater than 90% as an acceptable detection rate. However, our data shown in Table 2 illustrated L210W, unlike other alleles, had only a call rate per allele equal to 80.5%. Strictly, we will not accept the detection for this allele as completely successful and thus a redesign of the extension primers for detecting L210W will be required in future. In terms of the viral load required for our assay, we found that 4 independent specimens with viral load less than 500 copies/ml reached a call rate per sample higher than 90%. This result indicated the potential utility of this assay to detect mutations at early phase of viral rebound.

Apart from the high concordance rate between this MALDI-TOF MS based assay and the conventional sequencing, this assay was better than the conventional sequencing due to lower concentration of PCR products was required per reaction as determined by a two-fold serial dilutions approach (data not shown). In addition, this platform is capable of analyzing two 384-format chips (sufficient for total 126 samples plus two no template controls in each chip) in a single run. Given the high-throughput nature of this assay, results of hundreds of samples could be obtained within 36 hours including viral RNA extraction and nested PCR amplification. Moreover, this assay greatly reduced the labor required as only three steps including neutralization of unincoporated dNTPs, a PCR based iPLEX Gold reaction and desalting of extension products were needed before data analysis.

Another advantage of this assay over conventional sequencing is that this system is automated and easy to be operated by following the user manual once the extension primers were designed. Since genotyping results are readily available once the assay is finished, even individuals with basic training in bioinformatics can interpret the results easily and in a time saving manner.

Despite these advantages, both assays were estimated to have similar sensitivity for detecting the variants in total viral population as mentioned. Although the cost per sample, including the cost of viral RNA extraction and nested PCR amplification, for this MALDI-TOF MS based assay (RMB 80.00 per sample) is considerably lower than that for commercially available genotypic assays, such as ViroSeq (RMB 1089 per sample) and TruGene (RMB 1344 per sample) [8], a major limitation for implementing this assay in clinical settings and surveillance purpose in China is that the cost per sample for in-house sequencing assay is cheaper. To a certain extent, the higher cost has limited the number of mutations detected in our system. Fortunately, once the cost reduced, more drug resistance mutations of biological significance can be incorporated into the current assay by adding extension primers designed by designated computer program.

Recent advances in next generation sequencing, such as ultra-deep sequencing have significantly increased the sensitivity for detecting the low level drug resistant variants and permitted the quantification of them. However, these assays are not suitable for routine surveillance in developing countries such as China due to expensive cost and high technical content [33,34].

Similar to other genotypic assays for detecting HIV-DR mutations, this assay can only analyze the known resistance mutations and fail to provide sequence data. Also, the sensitivity for detecting low abundance variants is dependent on the number of viral templates provided for cDNA synthesis and PCR amplification [35,36]. In addition to the above limitations, our assay is a qualitative assay and cannot be used for the quantification of drug resistance mutations, although the Sequenom massARRAY® system is capable to quantify drug resistance mutations in patient specimens.

Since HIV-1 with drug resistance mutations against different categories of antiretroviral drugs has been identified, our future studies will be focusing on designing the multiplex assays for detecting the drug resistance mutations against the protease inhibitors, integrase inhibitors as well as entry inhibitors based on this Sequenom MassARRAY® system. The feasibility to establish a MALDI-TOF MS based assay, which can detect major HIV-DR mutations against all categories of antiretroviral drugs will also be investigated in future.

## Conclusions

Although the assay reported in this study was capable to detect drug resistance mutations for HIV-1 CRF01_AE, CRF07_BC, subtype B and other strains, its practical application in clinics was limited by the number of mutations detected at this moment. In conclusion, we provided a proof of concept in which it is possible to establish a simple, rapid, broadly reactive and automated high-throughput platform for multiplex detection of major HIV-DR mutations towards the commonly used NRTIs and NNRTIs in China using Sequenom MassARRAY® system.

## Supporting Information

S1 Fig Sequences and molecular weights (MW) of unextended primers (UEP) and extended primers (EP). (PDF)

## Abbreviations

HAART: Highly active anti-retroviral therapy
HIV-DR: HIV-1 drug resistance
MALDI-TOF MS: Matrix-assisted laser desorption ionization-time of flight mass spectrometry
NFATP: National Free Antiretroviral Treatment Program
NRTIs: Nucleoside reverse transcriptase inhibitors
NNRTIs: Non-nucleoside reverse transcriptase inhibitors
