# Supplementary material for: Rapid and Simultaneous Detection of Major Drug Resistance Mutations in Reverse Transcriptase Gene for HIV-1 CRF01_AE, CRF07_BC and Subtype B in China Using Sequenom MassARRAY® System
Source: PLoS One. 2016 Apr 19;11(4):e0153641. doi: 10.1371/journal.pone.0153641 (PMC4836728; doi:10.1371/journal.pone.0153641)
Supplement: S1 Fig — (PDF) [file pone.0153641.s001.pdf]

# Supplementary table 1

## Suppl. table 1. Sequences and molecular weights (MW) of unextended primers (UEP) and extended primers (EP)

| | SNP_ID | Relevant Drug resistance mutations | UEP_DIR | UEP Concentration (µM) | UEP Sequence | UEP MW (Da) | EP 1 (CALL) | EP 1 (Da) | EP 2 (CALL) | EP 2 (Da) | EP 3 (CALL) | EP 3 (Da) | EP 4 (CALL) | EP 4 (Da) |
|---|---|---|---|---|---|---|---|---|---|---|---|---|---|---|
| Assay 1 | 850RT4 | K101E/Q/P | F | 7 | CGCATCCAGCAGGTTTA | 5170.4 | C | 5417.6 | A | 5441.6 | G | 5457.6 | | |
| | 20563RT29 | T215F/Y | F | 7 | cTGTTGAGGTGGGGATTT | 5616.6 | A | 5887.9 | T | 5943.7 | | | | |
| | 581RT2 | K65R | F | 7 | CCAGTATTTGCCATAAAGA | 5795.8 | A | 6067 | G | 6083 | | | | |
| | 925RT1 | M41L | R | 14 | AAATTTTTCCTTCCTTTTCCA | 6272.1 | T | 6543.3 | C | 6559.3 | A | 6599.2 | | |
| | 885RT6 | K103N/S | F | 14 | caCCAGCAGGATTAAAAAAGA | 6465.3 | A | 6736.5 | G | 6752.5 | | | | |
| | 816RT25 | G190A | F | 14 | TACATGGATGACTTGTATGTAG | 6804.4 | C | 7051.6 | G | 7091.7 | | | | |
| Assay 2 | 20563RT26 | L210W | R | 7 | TGGTAAATCCCCACCTC | 5090.3 | G | 5337.5 | T | 5361.5 | | | | |
| | 885RT1 | M41L | R | 7 | ATTTTTCCTTCCTTTTCCA | 5645.7 | T | 5916.9 | C | 5932.9 | A | 5972.8 | | |
| | 975RT5 | K101E/Q/P | F | 14 | caACACCCAGCAGGGTTAA | 5790.8 | C | 6038 | A | 6062 | | | | |
| | 816RT11 | K103N/S | F | 14 | CCGGCAGGTTTAAAAAAGAA | 6183.1 | C | 6430.2 | A | 6454.3 | T | 6510.2 | | |
| | 816RT20 | M184V | R | 14 | ATCCTACATACAAGTCATCCA | 6318.1 | G | 6565.3 | A | 6645.2 | | | | |
| Assay 3 | 816RT26 | L210W | R | 7 | GTAGTAAATCCCCAGCTC | 5443.6 | G | 5690.7 | T | 5714.8 | | | | |
| | 850RT5 | K101E/Q/P | F | 7 | CCGCATCCAGCAGGTTTAA | 5772.8 | C | 6020 | A | 6044 | | | | |
| | 975RT4 | K101E/Q/P | R | 14 | aTGTCACTGATTTTTGCTTTT | 6383.2 | G | 6630.3 | C | 6670.4 | A | 6710.3 | | |
| | 925RT9 | G190A | F | 14 | ACATGGATGATTTGTATGTAG | 6515.3 | C | 6762.4 | G | 6802.5 | | | | |
| Assay 4 | 19RT29 | T215F/Y | F | 7 | TGTTGAGGTGGGGATTT | 5327.5 | A | 5598.7 | T | 5654.6 | | | | |
| | 22326RT2 | K65R | R | 7 | CCATTTGGTGCTGTCCTTT | 5751.7 | G | 5998.9 | A | 6078.8 | | | | |
| | 885RT2 | K65R | R | 14 | CTCCACTTAGTACTGTCTTTC | 6298.1 | G | 6545.3 | A | 6625.2 | | | | |
| | 816RT10 | K103N/S | R | 14 | CATCTAGTACTGTTACTGATTTT | 6994.6 | G | 7241.8 | A | 7321.7 | | | | |
| Assay 5 | 885RT7 | K103N/S | F | 7 | CCAGCAGGATTAAAAAAGAA | 6176.1 | C | 6423.3 | A | 6447.3 | G | 6463.3 | T | 6503.2 |
| | 830RT26 | L210W | R | 14 | TGGAGTAGTAAATCCCCACTTC | 6694.4 | G | 6941.6 | T | 6965.6 | | | | |
| Assay 6 | 581RT29 | T215F/Y | F | 7 | TATTGAGCTGGGGATTT | 5271.4 | A | 5542.6 | T | 5598.5 | | | | |
